# Assessment of prevalence, knowledge of polycystic ovary syndrome and health-related practices among women in klang valley: A cross-sectional survey

**DOI:** 10.3389/fendo.2022.985588

**Published:** 2022-08-29

**Authors:** Jia Ean Goh, Muhammad Junaid Farrukh, Fazlollah Keshavarzi, Chuan Sheng Yap, Zikria Saleem, Muhammad Salman, Diana Laila Ramatillah, Khang Wen Goh, Long Chiau Ming

**Affiliations:** ^1^ Faculty of Pharmaceutical Sciences, UCSI University, Kuala Lumpur, Malaysia; ^2^ Department of Pharmacy Practice, Faculty of Pharmacy, Bahauddin Zakariya University, Multan, Pakistan; ^3^Institute of Pharmacy, Faculty of Pharmaceutical and Allied Health Sciences, Lahore College for Women University, Lahore, Pakistan; ^4^ Pharmacy Faculty, Universitas 17 Agustus 1945, Jakarta, Indonesia; ^5^ Faculty of Data Science and Information Technology, INTI International University, Nilai, Malaysia; ^6^ PAP Rashidah Sa’adatul Bolkiah Institute of Health Sciences, Universiti Brunei Darussalam, Gadong, Brunei

**Keywords:** polycystic ovary syndrome (PCOS), prevalence, knowledge, health-related practices, Malaysia

## Abstract

**Background:**

Polycystic ovary syndrome (PCOS) is a common metabolic and reproductive disorder affecting women of childbearing age. Its symptoms associated with androgen excess and menstrual abnormalities have great impact on the quality of life in women with PCOS. Data on the knowledge of PCOS and health-related practices among Malaysian women is scarce. This study aimed to determine the prevalence, knowledge and health-related practices of PCOS among women in Klang Valley, Malaysia.

**Method:**

A descriptive cross-sectional study was conducted among women in Klang Valley, Malaysia using a self-administered questionnaire. Participants were conveniently recruited through online platforms. Questionnaire consisted of four sections. The knowledge scores ranged from 0-20 where < 10 was classified as poor knowledge. Health-related practices scores ranged from 10-50, with score <30 was classified as poor practice. Descriptive statistics was used to report demographic characteristics. Inferential statistics was used to report the differentiation, association, and correlations of the variables.

**Results:**

A total of 410 respondents participated in this survey. The finding revealed that 43 (10.49%) respondents had medical diagnosis of PCOS, 11 (2.68%) were diagnosed with PCOS based on signs and symptoms, and 135 (32.93%) were suspected with PCOS. Nearly half of the respondents had poor knowledge (47.30%) and poor practice (47.60%) of PCOS. Also, 46 (11.22%) respondents reported abnormal scalp hair loss and 30 (7.32%) respondents had diabetes. Educational levels and PCOS history were significantly associated with PCOS knowledge. Respondents with healthcare related educational background demonstrated good knowledge (p<0.01). Majority of respondents (n= 328, 80%) were unable to control their diets on weekends. Married participants and aged between 40 to 45 women showed better practice of PCOS.

**Conclusion:**

Nearly half of the respondents had poor knowledge and health-related practices towards PCOS. Women with suspected or diagnosed PCOS should seek immediate medical help as early diagnosis and treatment for PCOS are beneficial in improving their quality of life.

## Background

Polycystic Ovary Syndrome or polycystic ovarian syndrome (PCOS) was originally called “Stein-Leventhal syndrome” and later it was termed polycystic ovary disease (PCOD) before the name of PCOS (1). It is a heterogeneous, endocrine and metabolic disorder characterized by ovarian cysts, oligo- or anovulation, and hyperandrogenism that severely affects the lives of women of childbearing age (2–4).

The exact cause of PCOS is still unclear, but researchers believe that the disorder is primarily related to hyperandrogenism and hyperinsulinemia, which are associated with insulin resistance. These two elevated hormones regulate ovarian function and interfere with the normal functioning of other hormones that regulate menstruation. A constant hormonal imbalance in the body impairs the functioning of the ovaries, leading to the formation of cysts inside the ovarian sac. This is where the name polycystic ovary syndrome comes from (3). Other causes of PCOS include stress, obesity, genetics, lifestyle, and prenatal factors that cause PCOS to vary degrees of influence (3, 5–12).

PCOS is commonly affected by hyperandrogenism and hyperinsulinemia which additionally result in different complicated dysfunctional mechanisms in the body. Due to those internal dysfunctions, PCOS causes unpleasant symptoms such as oligomenorrhea/amenorrhea, infertility/early miscarriage, hirsutism, acne, androgenetic alopecia, and acanthosis nigricans (13).

Having PCOS will lead to reproductive challenges such as infertility, late menopause, and even endometrial cancer. PCOS also puts the women at a higher risk of metabolic syndrome due to the presence of risk factors such as central obesity, high blood pressure, atherosclerotic dyslipidemia, and insulin resistance. Because of these risk factors, long-term consequences such as type 2 diabetes, heart disease, sleep apnea, and psychological problems including anxiety and depression are common in women with PCOS (14). Therefore, an early diagnosis and treatment of PCOS are crucial to prevent future long-term complications and reduce the healthcare burden.

For the diagnosis of PCOS, the Rotterdam criteria are widely used worldwide, and its use is recommended by the Endocrine Society in 2013, the American Academy of Family Physicians (AAFP) Guidelines in 2016, and the International Evidence-based Guideline for the assessment and management of polycystic ovary syndrome 2018. These criteria stated that women must have at least two out of the three criteria for diagnosing PCOS, including the presence of oligo-/anovulation, clinical or biochemical hyperandrogenism and/or ovarian cysts. Other possible hormonal disorders should also be excluded (15–17). It meets the diagnostic requirements set by the Centers for Disease Control and Prevention (CDC) (5). In addition to using the Rotterdam criteria for the diagnosis of PCOS, a thorough evaluation of the patient’s medical history, physical examination, basic laboratory tests and comorbidity risk assessment is recommended without ultrasound and other types of imaging test (15, 18, 19).

PCOS treatment should be individualized according to the patient’s clinical presentations and treatment should be directed toward achieving the desire to conceive. According to American Family Physician (AAFP) Guideline 2016 and the Australian Family Physician (AFP) Guideline 2012, if pregnancy is desired, first-line medications like clomiphene or letrozole or second-line drug metformin is recommended to treat infertility or anovulation. *In vitro* fertilization (IVF) is one of the common treatments for women with PCOS but presents multiple challenges ranging from a poor to an exaggerated response, poor egg to follicle ratio, poor fertilization, poor blastocyst conversion and ovarian hyperstimulation syndrome (20).

If pregnancy is not desired, the first-line therapy for ovulatory dysfunction should be hormonal contraceptives such as hormonal intrauterine devices (IUDs) or oral contraceptives, followed by metformin as the second-line therapy. Patients can be prescribed topical cream, benzoyl peroxide for acne, electrolysis and phototherapy for hirsutism, metformin for insulin resistance, and lifestyle modifications for obesity to overcome the specific condition they are facing (15, 21, 22).

In 2020, the global prevalence of PCOS is between 2.2 to 48% (23, 24). Studies have shown a trend toward the increasing prevalence of PCOS since the late 1900s (23). Locally, a prevalence study at University Putra Malaysia in Malaysia found that the prevalence of PCOS among employees was 12.6% (25). Studies on prevalence, knowledge, and health-related practices were conducted in many countries but the data in Malaysia are lacking. Hence, this study aimed to determine the prevalence of PCOS, assess knowledge of PCOS and health-related practices among women in Klang Valley, Malaysia.

## Methods

### Study design and setting

This study was a descriptive cross-sectional study conducted from June 2021 to August 2021 among Malaysian women in Klang Valley, Malaysia. The participants were recruited conveniently from several social media platforms, such as Facebook, WhatsApp, Instagram, Messenger, Twitter, WeChat and Telegram.

### Sampling method and sampling size

Volunteers were recruited to participate in this study using convenience sampling techniques. Malaysian women between the ages of 18 and 45 and willing to participate were included in the survey.

The sample size was calculated using the online Raosoft Software^®^, a sample size calculator with a marginal error of 5%, 95% confidence level, and 50% response distribution. A minimum of 385 respondents was required in this study as shown below.

N=Z2P(1-P)/d2

Z= Z-score for 95% CI = 1.96

P (response distribution) = 50% = 0.5

d (margin of error) = 5% = 0.05

n = (1.96)2 x 0.5 (0.5)/(0.05)2

n = 0.9604/0.0025

n = 384.16

A total of 410 respondents completed the survey.

### Study tools

A pre-tested, self-guided, close-ended and structured questionnaire that adapted from two former studies was used for data collection (13, 26). The questionnaire consisted of four parts. The first section captured the demographic characteristics of the respondents. The second section consisted of 20 questions about PCOS knowledge. The third section consisted of 12 clinical assessment questions related to the prevalence of PCOS (13) and the last section of the questionnaire contained 10 questions regarding health-related practices (26).

The research tool was translated from English to Malay (Bahasa Melayu/Malaysia) by an accredited translating agency. The forward and backward translation of the questionnaire was performed by a registered proofreading company. Then, two bilingual scholars and a practicing pharmacist reviewed the translated forms. Copies of the questionnaire in both languages ​​are attached in the Appendix section.

### Scoring criteria

Mean cut-off points were used for both the knowledge section and the practice section, and the scoring criteria for the clinical evaluation were adapted from a former study in Pakistan (13). A total of 20 knowledge questions were posed and assessments were performed using a mean cut-off point of 11. Respondents with a knowledge score of less than 10 were categorized as poor knowledge and those with a knowledge score of 11 or higher were classified as good knowledge.

In the clinical evaluation section, a total of 12 questions were asked about the presence of signs and symptoms of PCOS. Respondents with three or fewer PCOS symptoms were not diagnosed with PCOS, while respondents with four to eight symptoms were suspected of having PCOS. On the other hand, the diagnosis of PCOS is made if a respondent is having more than eight symptoms of PCOS (13)..

Moving on to the section on health-related practices, the questionnaire asked ten questions assessing respondents’ health-related practices. A 5-point Likert scale was utilized to rate respondents’ psychometric responses to each of the ten questions. The 5-point Likert scale measures the level of agreement with a statement and is typically on a 5-point scale including never, rarely, sometimes, usually, and always. A mean cut-off value of 31 was applied. This means that the respondents who scored 30 or less from these ten questions were categorized as having poor health-related practices whereas the remaining respondents with a health-related practice score of at least were classified as people having good health-related practices.

### Validation of questionnaire and pilot study

For face and content validation, the questionnaire was reviewed by a panel of experts comprising of researchers, physicians, academicians, and pharmacists. These experts reviewed the questionnaire based on content relevance, clarity, simplicity, and ambiguity. After revising the questionnaire considering comments of the expert-panel, a pilot study was carried out among 30 subjects to ensure the reliability of the questionnaire formulated.

The internal consistency was calculated by Cronbach’s alpha coefficient which was 0.861 in the knowledge section and 0.871 in the health-related practice section.

### Data collection

Ethical approval was obtained from the UCSI university ethics committee (Ref. no. IEC-2020-FPS-043). Data was collected from the participants through online platforms. Informed consent was provided along with the survey form. Before the survey begins, participants were required to fill their initials as the signature to indicate they agreed to participate in the study and the information given was accurate to the best of their knowledge. All participants were informed about the purpose of the study.

### Data analysis

Statistical analysis was performed using the IBM SPSS Statistics Version 23.0. The resulting data were analyzed using both descriptive and inferential analysis. Descriptive analysis included percentages, means and standard deviations used to report demographic characteristics and knowledge sections. For inferential analysis, significant differences between variables were analyzed using parametric tests such as independent t-tests, one-way ANOVA and cross-tabulation analysis.

A total of 20 knowledge items were given a score from 0 to 20. Knowledge with scores ranging from 0 to 10 was categorized as poor knowledge, and scores from 11 to 20 were classified as good knowledge. Each element of the knowledge was presented with three options (yes, no, don’t know), with one correct and two incorrect answers. A correct answer was given 1 point, and an incorrect answer was given 0 point. Those who answered all correctly received a total of 20 points.

Meanwhile, in the practice section, only 10 items were used on a 5-point Likert scale. For each question, 5 points were awarded for the most appropriate and correct answer and 1 point for the lowest answer. Likert scales for the practice section included never, rarely, sometimes, usually, and always. Potential scores for this section range from 10 to 50, with 10 to 30 classified as poor practices and 31 to 50 as good practices.

## Results

A total of 410 respondents participated in this survey. The respondents were having an average age of 25.92 years old (SD = 6.287, range 18-45) while the age group 21 to 29 overtook respondents at a rate of 67.3% (n = 276). The majority of the respondents were Chinese (n = 281, 68.5%), followed by Malays (n = 66, 16.1%), Indians (n = 51, 12.4%) and representatives of other ethnicities including Iban, Sikhs, Dayaks, Chindians, Sino, Eurasian, and Portuguese (n = 12, 2.9%). Additionally, the majority of the respondents were not related to healthcare (n = 224, 54.6%).

The mean ± SD of knowledge, clinical evaluation and health-related practices scores in this study were 10.31 ± 5.428 (51.55%), 3.49 ± 2.438 (29.08%), and 31.25 ± 6.886 (62.5%) correspondingly. In this study, 11 (2.68%) respondents were diagnosed with PCOS based on signs and symptoms, 135 (32.93%) respondents were suspected of PCOS, and 43 (10.49%) were medically diagnosed with PCOS. There were 194 (47.30%) respondents depicted poor knowledge and 195 (47.60%) respondents followed poor health-related practices.

The details of overall knowledge, health-related practice scores and respondents’ sociodemographic characteristics are portrayed in **Table 1**. The clinical evaluation is shown in **Table 2**. There was significant difference of mean knowledge scores between races, educational level, area of study involved, and history of PCOS. *Post hoc* analysis showed that this difference was larger between Chinese and Indians. Mean practice scores was significantly different between age groups, marital status, monthly household income, and body mass index categories. The difference was larger between single and married women.

**Table 1:** Summary of knowledge and health-related practice scores with sociodemographic characteristics.

Variables	Categories	Mean score ± SD	
		Knowledge	Practices
Age	18 - 20	8.31 ± 6.031	29.64 ± 7.151
	21 – 29	10.66 ± 5.420	30.72 ± 6.993
	30 – 39	10.29 ± 5.300	32.87 ± 5.743
	40 - 45	9.68 ± 4.337	35.12 ± 6.412
	p value	0.078 ^b^	0.001 ^b **^
Race	Malay	10.33 ± 5.094	31.02 ± 6.779
	Chinese	10.00 ± 5.437	31.19 ± 6.952
	Indian	12.73 ± 4.912	32.08 ± 6.161
	Other	7.54 ± 6.346	30.54 ± 9.024
	p value	0.002 ^b **^	0.831 ^b^
Marital status	Single	10.23 ± 5.511	30.73 ± 7.007
	Married	10.91 ± 5.026	33.84 ± 5.489
	Divorced	8.50 ± 4.848	33.33 ± 7.737
	p value	0.471 ^b^	0.003 ^b **^
Educational level	Primary school	3.00	22.00
	Secondary school	8.69 ± 5.218	28.85 ± 6.162
	Diploma/Pre-U/STPM/A-level	7.95 ± 5.769	30.95 ± 6.862
	Degree	10.64 ± 5.379	31.19 ± 6.887
	Master	10.76 ± 4.854	31.86 ± 6.665
	PhD	11.19 ± 5.845	33.88 ± 7.830
	p value	0.024 ^b **^	0.288 ^b^
Area of study involved	Non-healthcare related	8.64 ± 5.306	30.90 ± 7.130
	Healthcare-related	12.32 ± 4.875	31.67 ± 6.575
	p value between groups	0.000 ^a **^	0.260 ^a^
Employment status	Unemployed	11.07 ± 4.949	30.87 ± 5.553
	Employed	10.58 ± 5.309	31.41 ± 6.971
	Self-employed	8.76 ± 5.380	33.40 ± 6.171
	Student	10.18 ± 5.630	30.73 ± 6.952
	Housewife	12.00 ± 1.871	36.80 ± 6.648
	Retired	12.00	33.00
	p value	0.638 ^b^	0.221 ^b^
Monthly household income	<RM1,000	8.69 ± 4.662	26.69 ± 5.329
	RM1,000 – RM3,000	9.64 ± 6.076	31.16 ± 7.382
	RM3,001 – RM5,000	9.90 ± 5.398	30.27 ± 7.255
	>RM5,000	10.84 ± 5.104	31.81 ± 6.520
	p value	0.147 ^b^	0.037 ^b **^
Districts and areas	Kuala Lumpur	10.37 ± 5.725	30.69 ± 7.217
	Putrajaya	9.00 ± 8.099	30.67 ± 5.888
	Selangor: Gombak	10.33 ± 4.592	30.56 ± 6.336
	Selangor: Hulu Langat	9.77 ± 5.424	31.23 ± 7.095
	Selangor: Klang	12.50 ± 3.954	31.85 ± 8.312
	Selangor: Kuala Langat	10.90 ± 5.131	29.50 ± 4.197
	Selangor: Petaling	10.42 ± 5.470	32.39 ± 7.165
	Selangor: Sepang	7.36 ± 4.651	30.00 ± 4.489
	Other	10.95 ± 5.490	31.14 ± 4.570
	p value	0.353 ^b^	0.703 ^b^
Medically diagnosed with PCOS	No	9.90 ± 5.457	31.09 ± 6.850
	Yes	13.79 ± 3.681	32.65 ± 7.111
	p value	<0.001 a ^**^	0.159 ^a^
Any underlying health conditions	No	10.19 ± 5.540	31.18 ± 6.930
	Other	11.40 ± 4.150	31.95 ± 6.504
	p value	0.182 ^a^	0.500 ^a^
BMI category	<18.5 kg/m^2^	9.56 ± 5.993	29.31 ± 6.437
	18.5 kg/m^2^– 24.9 kg/m^2^	10.38 ± 5.286	32.21 ± 6.736
	25.0 kg/m^2^– 29.9 kg/m^2^	10.46 ± 5.167	30.93 ± 7.039
	≥30 kg/m^2^	11.60 ± 5.315	29.83 ± 7.813
	p value	0.337 ^bf^	0.005 ^b **^

**indicates that p < 0.05 is significant; a = Independent t-test; b = One-way ANOVA.

**Table 2:** Clinical evaluation of PCOS.

Variables	Categories	Frequency (n)	Percentages (%)
Clinical evaluation of PCOS	Not diagnosed	221	53.90
	Suspected	135	32.93
	Currently diagnosed	11	2.68
	Previously diagnosed	43	10.49

A detailed breakdown of respondents’ response to knowledge questions is portrayed in **Table 3**. There were 215 (52.40%) respondents unaware that PCOS increased androgen levels. Of them, 256 (62.40%) were aware of the presence of small multiple cysts in the ovaries of patients diagnosed with PCOS. Also, 299 (72.90%) respondents did aware that irregular menstruation is a symptom of PCOS. However, 255 (62.20%) of them did not know that prediabetes condition may lead to PCOS. Furthermore, 320 (78.00%) respondents were reported that they did not know PCOS may cause heart disease. There were 262 (63.90%) respondents unaware that PCOS symptoms can be improved by taking symptomatic treatment such as clomiphene and letrozole.

**Table 3:** Responses of respondents to knowledge questions.

Questions for Knowledge of PCOS	Number of Responses (%)	
	No/Incorrect	Yes/Correct
Have you heard about the term called “polycystic ovary syndrome (PCOS)”?	159 (38.80%)	251 (61.20%)
Have you heard about the androgen (male) hormone? E.g. testosterone	66 (16.10%)	344 (83.90%)
In PCOS, there is an increased level of androgen level.	215 (52.40%)	195 (47.60%)
Patients suffering from PCOS have small multiple cysts in their ovaries.	154 (37.60%)	256 (62.40%)
Obesity may cause PCOS.	225 (54.90%)	185 (45.10%)
Prediabetes condition (due to decreased insulin action in the body) may cause PCOS.	255 (62.20%)	155 (37.80%)
Irregular or absence of menstrual (periods) cycle is a symptom of PCOS.	111 (27.10%)	299 (72.90%)
An unusual amount of hair growth on different body parts (upper lip, chin, abdomen, breast, thighs etc.) is a symptom of PCOS.	188 (45.90%)	222 (54.10%)
Severe acne problem during the menstrual (periods) cycle is a symptom of PCOS.	220 (53.70%)	190 (46.30%)
Hair loss from the scalp more than normal is a symptom of PCOS.	256 (62.40%)	154 (37.60%)
PCOS diagnosis can be confirmed by vaginal ultrasound.	176 (42.90%)	234 (57.10%)
A specific blood test can be used for the diagnosis of PCOS.	231 (56.30%)	179 (43.70%)
PCOS may lead to diabetes (long-term high blood sugar level).	280 (68.30%)	130 (31.70%)
PCOS may lead to heart diseases.	320 (78.00%)	90 (22.00%)
PCOS may lead to infertility (inability to have children) or reduced fertility (reduced chance to get pregnant).	98 (23.90%)	312 (76.10%)
PCOS may lead to anxiety and depression.	148 (36.10%)	262 (63.90%)
Hormonal therapy (oral contraceptives, hormone intrauterine device etc) may be used to treat PCOS.	170 (41.50%)	240 (58.50%)
Anti-diabetic medications (metformin) may be used to treat PCOS.	294 (71.70%)	116 (28.30%)
Symptomatic treatment (clomiphene, letrozole, acne topical cream, spironolactone etc) may be given to relieve the symptoms of PCOS.	262 (63.90%)	148 (36.10%)
Surgery may be used to remove ovarian cysts.	144 (35.10%)	266 (64.90%)

A total of 159 (38.8%) respondents usually consumed high-fiber foods in their daily diets while 151 (36.8%) of them sometimes consumed high-fiber foods in their diets. There were 132 (32.2%) respondents who claimed that they sometimes read nutrition labels, 128 (31.2%) respondents usually read nutrition labels, and 55 (13.4%) who always do that. Yet, there were 19 (4.6%) respondents who never read nutrition labels in their daily lives. The majority of the respondents that occupied 148 (36.1%) sometimes incorporated low-fat foods into their diets whilst 128 (31.2%) usually incorporated low-fat meals into their diets. Additionally, 126 (30.7%) of them rarely control their diets on weekends. Responses of respondents to PCOS practice statements are shown in **Table 4**. There was no significant difference of mean knowledge score between the BMI categories. However, a significant difference of mean practice score was found between the BMI categories. *Post-hoc* analysis revealed that participants under Obese category (BMI≥30 kg/m^2^) were more likely to have poor practice.

**Table 4:** Responses of respondents to health-related practice statements.

Questions for Practices	Number of Respondents (%)				
	Never	Rarely	Sometimes	Usually	Always
How often do you read nutrition labels?	19 (4.6%)	76 (18.5%)	132 (32.2%)	128 (31.2%)	55 (13.4%)
I incorporate low-fat foods into my diet.	21 (5.1%)	76 (18.5%)	148 (36.1%)	128 (31.2%)	37 (9.0%)
I incorporate low salt foods into my diet.	23 (5.6%)	85 (20.7%)	129 (31.5%)	118 (28.8%)	55 (13.4%)
I eat 5 servings of fruits and vegetables per day.	43 (10.5%)	141 (34.4%)	132 (32.2%)	64 (15.6%)	30 (7.3%)
I decrease the amount of refined sugar in my diet.	12 (2.9%)	63 (15.4%)	140 (34.1%)	131 (32.0%)	64 (15.6%)
I eat more high-fibre foods.	6 (1.5%)	44 (10.7%)	151 (36.8%)	159 (38.8%)	50 (12.2%)
I eat smaller portions at dinner.	19 (4.6%)	67 (16.3%)	109 (26.6%)	133 (32.4%)	82 (20.0%)
I exercise 30 minutes 5 days a week.	87 (21.2%)	133 (32.4%)	84 (20.5%)	66 (16.1%)	40 (9.8%)
I control my eating on weekends.	83 (20.2%)	126 (30.7%)	119 (29.0%)	54 (13.2%)	28 (6.8%)
It is easy for me to eat a healthy diet.	28 (6.8%)	83 (20.2%)	135 (32.9%)	102 (24.9%)	62 (15.1%)

There was no significant association between the knowledge of PCOS and the health-related practices toward PCOS. Although most of the respondents were having good knowledge and practices, yet 108 (55.40%) of them had good knowledge but poor practices. The detailed association is shown in **Table 5**.

**Table 5:** Association between knowledge and health-related practices.

Variable		Knowledge of PCOS		p-value
		Poor	Good	
Health-related practice	**Good**	107 (49.80%)	108 (50.20%)	0.297
**Poor**	87 (44.60%)	108 (55.40%)

## Discussion

In 2020, the worldwide prevalence of PCOS had been reported to range from 2.2% to 48%, including countries such as India, Australia, USA, Iran and China (23, 24). In Pakistan, a previous study that used the same questionnaire reported 5.5% of those with previously diagnosed people and 3.5% of those with currently diagnosed people (13). The prevalence rate reported in this study was 10.49% for those who were previously diagnosed with PCOS and 2.48% for those who are currently diagnosed based on the reported signs and symptoms. This study also found that 32.93% of the respondents were suspected of PCOS.

Based on these data, our study suggested that Malaysians had a lower prevalence of PCOS than the global reported prevalence rate that was as high as 48% (23, 24). This statement is further supported by a comparable previous prevalence study conducted at University Putra Malaysia which reported a similar prevalence rate of 12.6% in 2019 (25).

Differences in prevalence were associated with different study populations (13). In addition, the prevalence rate varied from study to study because three diagnostic criteria were used to diagnose PCOS, including the Rotterdam criteria, the NIH criteria, and the AE-PCOS criteria. Among these diagnostic tools, the Rotterdam criteria is internationally recognized and preferred but has the highest prevalence compared to other diagnostic tools (27).

Evidence from this study suggested that race, education level, the field of study, and history of PCOS diagnosis were significantly affecting the public knowledge of PCOS. Our study showed that Indians significantly had better knowledge of PCOS than Chinese. This may be due to the smaller sample size of Indians than the Chinese. Further studies using uniform sample sizes from different ethnicities may be needed to confirm this significant difference between Chinese and Indians.

Besides, as suggested by the data, knowledge of PCOS was significantly influenced by educational level. This finding indicated that the higher the educational level of Malaysian women, the higher their knowledge of PCOS. This finding was consistent with other studies in Saudi Arabia as well as in Jordan that also explained the positive relationship between educational levels and PCOS knowledge (28, 29).

Also, our study suggested that the field of study had a significant impact on knowledge about PCOS. In this study, respondents with healthcare-related backgrounds showed a higher level of knowledge than respondents without it. This was because healthcare professionals are the primary information source of PCOS as reported by evidence (30) and they are more specialized in the healthcare field than the general public who less keep in touch with healthcare information. Hence, Malaysians with healthcare-related backgrounds had a higher level of PCOS knowledge than the general public.

Apart from that, our finding suggested that respondents with the previous diagnosis of PCOS by medical professionals were more knowledgeable about PCOS than those who did not. This finding was consistent with another comparable study comparing knowledge of PCOS in women with PCOS and women without. In that study, the authors reported a higher knowledge level about female reproductive hormones among women with PCOS than healthy women (31). Another study that assessed the knowledge level of PCOS in women diagnosed with PCOS also reported a higher level of PCOS knowledge in women after diagnosis of PCOS than before diagnosis (32).

Since medical professionals are the primary information source of PCOS (30), the respondent who had been formally diagnosed with PCOS had better knowledge of PCOS due to the fact they acquired accurate information regarding PCOS from the medical professionals upon diagnosis of PCOS and often in the course of the follow-up sessions. As limited data about PCOS is available in Malaysia, the general public who much less common consulting physicians or medical professionals hard to have knowledge of PCOS than those who had the right supply of PCOS information.

On the other hand, data of our study suggested that health-related practices were significantly influenced by age, marital status, monthly family income, and body mass index. This indicated that the older the age Malaysian, the better the health-related practices towards PCOS. This is because as people grow older, they usually will experience more health issues and be able to learn from their past medical experience as compared to the younger generation who have less experience.

In addition, married respondents had better health-related practices as compared to single respondents. This was in line with several studies that reported greater psychological and physical health among married adults with happy marriages as compared to non-married adults (33, 34). This could be due to the changing health behaviors in married people with happy relationships as compared to those unmarried that rarely change without influences from partners or others (34).

Our finding also suggested that people with higher monthly household incomes will have a greater chance of having better health-related practices. A study showed that with a higher income, a person will have a greater ability to buy health-promoting goods and engage in social life in ways that enable people to be healthy. The authors also explained that people who live in poor environments may have a greater possibility to engage in unhealthy behaviors (35). Also, people with higher monthly household incomes may have greater free time to self-care as they require lesser time to earn money. They will also have more opportunities for self-development and access to healthcare facilities.

Generally, our study showed that the majority of the respondents had good knowledge of PCOS with a percentage of 52.70%. This data contradicted the theory put forward by most studies that the public was unaware of the health problems associated with PCOS (29, 31, 32, 36–39). Although this finding opposed the theory proposed by the majority of the studies, there was a study showing a good level of knowledge among the public with higher secondary education (28) and average knowledge among students (30). Since the respondents in our study were mainly students, they may have better knowledge of PCOS as compared to the other studies that were studied on the general public.

The majority of the respondents in our study showed good health-related practices. As most of the respondents were came from households having higher income levels, the respondents may have better health-related practices due to the higher possibility of satisfying health-related needs and the positive impact of high-income levels on health-related practice.

A significant difference of mean practice score was found between the BMI categories. *Post-hoc* analysis revealed that participants under Obese category (BMI≥30 kg/m^2^) were more likely to have poor practice. One of the main findings of the data suggested that the knowledge level of PCOS was not significantly associated with health-related practices towards PCOS. This indicated that even a person has good knowledge of PCOS, it does not necessarily indicate that the same person will also have good health-related practices towards PCOS. Similarly, a study in the United States of women with and without PCOS found that women with PCOS performed worse in health-related practices, despite being knowledgeable about PCOS (31). We speculated that this may be due to differences in individual thoughts and attitudes toward PCOS. Healthcare professionals should conduct educational campaigns specially targeting these vulnerable groups who are overweight and obese with higher risk of PCOS complication and motivate them on healthier lifestyle.

Last but not least, attitudes towards PCOS also played an important role in affecting the health-related practices among people who were having varying knowledge levels of PCOS. This point of view was because if a person has a negative attitude toward PCOS, she may not practice a good healthy lifestyle to reduce the risk of getting PCOS by following good health-related practices. If a person owns a positive attitude toward PCOS, the outcome will be vice versa.

## Limitations

This research was conducted online *via* multiple social media platforms, there is a possibility of biasness toward some underprivileged populations (aborigines/orang asli) that may not have access to social media. Another limitation found in this study is that the respondents may answer questions based on what they think is right instead of what they have been practicing in their daily lives. Data was collected by convenient sampling, which is non-probabilistic nature of the sampling strategy, which is vulnerable to selection bias (40).

## Conclusion

Nearly half of the respondents had poor knowledge and health-related practices towards PCOS. Most of the respondents were unaware that PCOS can lead to heart disease and the usefulness of symptomatic treatments like Clomiphene and Letrozole in enhancing PCOS symptoms. Women with suspected or diagnosed PCOS should seek immediate medical help as early diagnosis and treatment for PCOS are beneficial in improving their quality of life. These findings would help health policymakers to develop community-based awareness modules and India-specific management guidelines for early screening and a continuum of care for PCOS patients.

## Data availability statement

The raw data supporting the conclusions of this article will be made available by the authors, without undue reservation.

## Ethics statement

The studies involving human participants were reviewed and approved by the UCSI university ethics committee (Ref. no. IEC-2020-FPS-043). The patients/participants provided their written informed consent to participate in this study.

## Author contributions

MJF conceptualized the study and performed the analysis and interpretation of the data. JEG and DLR collected data and wrote the original manuscript. FK and CSY helped in creating our methodology and assisted in manuscript writing. ZS and MS assisted in literature review and questionnaire development. KYG and LCM reviewed the manuscript and assisted in discussion section. All authors have made an intellectual contribution to the work and have approved the final version of the manuscript for submission.

## Acknowledgments

Foremost, the authors would like to express gratitude to the Faculty of Pharmaceutical Sciences of UCSI University for approving this study. The authors also sincerely thank the public of Malaysia for spending their time by participating in the survey.

## Conflict of interest

The authors declare that the research was conducted in the absence of any commercial or financial relationships that could be construed as a potential conflict of interest.

## Publisher’s note

All claims expressed in this article are solely those of the authors and do not necessarily represent those of their affiliated organizations, or those of the publisher, the editors and the reviewers. Any product that may be evaluated in this article, or claim that may be made by its manufacturer, is not guaranteed or endorsed by the publisher.
